# Translaminar Pressure Difference and Ocular Perfusion Pressure in Glaucomatous Eyes with Different Optic Disc Sizes

**DOI:** 10.18502/jovr.v16i2.9080

**Published:** 2021-04-29

**Authors:** Natasha F. S. Cruz, Katia S. Santos, Mateus L. Matuoka, Niro Kasahara

**Affiliations:** ^1^Department of Ophthalmology, Irmandade da Santa Casa de Misericordia de Sao Paulo, Sao Paulo, Brazil; ^2^Santa Casa de Sao Paulo School of Medical Sciences, Sao Paulo, Brazil

**Keywords:** Cerebrospinal Fluid Pressure, Glaucoma, Ocular Perfusion Pressure, Optic Disc, Translaminar Pressure

## Abstract

**Purpose:**

Intracranial pressure (ICP) and ocular perfusion pressure (OPP) are both involved with the pathogenesis of glaucoma. The orbital ICP determines a retrolaminar counter pressure that is antagonistic to the intraocular pressure (IOP). The purpose of this study is to evaluate whether the translaminar pressure difference (TLPD) and the OPP varies in glaucoma patients with different optic disc sizes.

**Methods:**

In this university hospital-based, observational, cross-sectional clinical study, all patients underwent an ophthalmic evaluation. Blood pressure, height, weight, and the results of retinal nerve fiber layer examination with optical coherence tomography examination were recorded. TLPD and OPP were calculated for each patient using proxy algorithms to attain indirect surrogate parameter values. Patients' eyes were stratified into three quantiles according to optic disc sizes and the differences compared. Data from both eyes were used after using the appropriate correction for inter-eye dependency.

**Results:**

The sample consisted of 140 eyes of 73 patients with primary open-angle glaucoma and suspects. Patients with large disc size presented with higher TLPD as compared to those with average and small-sized discs (2.4 ± 4.5, 2.8 ± 3.8, and 3.7 ± 4.7 mmHg for first, second, and third tertile, respectively (*P* < 0.000). OPP did not vary according to the optic disc size.

**Conclusion:**

Glaucoma patients with larger optic discs have higher TLPD. The pathological significance of this finding warrants further investigation.

##  INTRODUCTION

Primary open-angle glaucoma (POAG) is a highly prevalent, sight-threatening, multifactorial disease. Its pathogenesis is associated with both mechanical and vascular factors. Mechanical factors including increased intraocular pressure (IOP) with posterior bulging of the cribriform blade, compression of the nerve fibers, and reduction of the retrograde and anterograde flow and vascular factors including decrease of perfusion pressure in the optic nerve head and deficiency in autoregulation leading to apoptosis of retinal ganglion cells and visual function loss may be involved.^[1]^

Recently, the intracranial pressure (ICP) was thought to be involved in the pathogenesis of POAG. Some clinical and population studies reported that glaucoma patients have a lower ICP compared to that of normal subjects.^[2,3]^ From an anatomical perspective, the ICP at the orbit level and the optic nerve tissue pressure determine the retrolaminar counter pressure which is antagonistic to the IOP. Thus, it may be part of the critical translaminar gradient or simply a translaminar pressure difference (TLPD). Presuming that there is a higher difference in the cribrosa translaminal pressure, a marked translaminar pressure gradient may damage the optic nerve, and therefore a low orbital ICP may be associated with the pathogenesis of glaucoma. There has been a debate as to whether this is an epiphenomenon or that there is an actual causal relationship between ICP and glaucoma.^[4]^

Several population-based and clinical studies support a strong association between ocular blood flow and the risk of POAG prevalence and progression.^[5,6]^ The underlying pathologic mechanism is related to the reduction in blood perfusion caused by impaired vascular autoregulation. The ocular perfusion pressure (OPP) is a physiologic function that delivers arterial blood to capillary bed for the eye tissues.^[7]^

Clinical variables such as TLPD and the OPP are potential players in the glaucoma optic neuropathy. Moreover, the optic disc size can vary substantially in the population.^[8,9]^ There is evidence suggesting that large optic discs may be more susceptible to glaucoma than small discs.^[10]^ Uncertainties regarding whether the TLPD and OPP differ according to the optic disc size and its influence in the glaucoma optic neuropathy exist. This cross-sectional, observational study aimed to assess whether TLPD and OPP vary in glaucoma patients and suspects according to the size of the optic discs.

##  METHODS

This cross-sectional, observational clinical study was approved by the Committee on Human Research of the institution. The participants were patients from the Glaucoma Service, Santa Casa de Misericordia of Sao Paulo Hospital. The study adhered to the tenets of the Declaration of Helsinki and its late amendments and the Resolution 466/12, National Council of Health, Brazilian Ministry of Health. After explaining the study procedures, all participants signed the informed consent.

### Study Population and Inclusion Criteria

The sample included patients who were diagnosed with POAG and met the following inclusion criteria: age > 40 years, any sex and ethnicity; no previous ocular lasers or incisional surgeries, except for cataract which occurred more than a year ago; optic disc with the presence of concentric increase or localized defect (notching) of the neural rim, disc hemorrhage, or a retinal nerve fiber layer (RNFL) defect; visual field defect characterized by at least three adjacent points on the pattern deviation map with *P* < 5% and one of the points with *P* < 1%, and/or pattern standard deviation (PSD) decreased with *P* < 5%, and/or glaucoma hemifield test (GHT) outside normal limits on a reliable exam. Perimetric examination with up to 20% of fixation loss and <15% of false positives and false negatives were considered reliable. Subjects with optic disc features of POAG and normal visual fields were included as suspects.

### Procedures

After a brief medical interview, patients participated in the study procedures. Demographic data including age, gender, ethnicity, and medical history (comorbidities and previous surgeries) were collected prior to the evaluations. Height (cm) was measured with the patient's back against the wall, without shoes, and feet together using a standard stadiometer. The body weight (kg) was measured on a calibrated manual platform scale with the patient wearing light clothing. The body mass index (BMI) of each participant was determined as the body mass divided by the square of the body height (kg/m${}^{2}$). Brachial arterial blood pressure was measured with the aneroid sphygmomanometer (Gurin Products, LLC, Tustin, CA, USA) using the right arm with the patient in a sitting position.

The participants received a complete ophthalmic examination which included measurement of visual acuity in the Snellen table with optical correction, anterior segment biomicroscopy with a slit lamp, tonometry with the Goldmann applanation tonometer (Haag-Streit AG, Switzerland) after taking a drop of fluorescein and proparacaine, gonioscopy with Goldmann goniolens (Ocular Instruments, Bellevue, Washington, USA), and optical disc evaluation with the 78D Volk lens (Volk Optical Inc., Mentor, OH, USA) after pharmacological mydriasis with tropicamide eye drops 0.5% and visual field examination. Computerized perimetry was performed with the HFV 750 (Carl-Zeiss Humphrey, Dublin, CA, USA), SITA standard program 24-2, with appropriate optical correction by a technician.

Optical coherence tomography (OCT) was performed with the OCT Angiography RTVueⓇ Avanti XR (Version 2015.1.0.90; Optovue Inc., Fremont, CA, USA). The OCT images were obtained at a rate of 26,000 A-scan/s and with a frame rate between 256 and 4096 A-scan/frame. This provided a high tissue resolution (depth resolution of 5.0 μm and transverse resolution of 15 μm). The acquisition of images in all patients followed the same procedure and was carried out by one technician. The retinal ganglion cells in the macular region were assessed using the Nerve Fiber Scan Protocol after pharmacologic dilation of the pupils. Images were excluded if the signal strength index (SSI) < 40; with overt decentration of the measurement circle location; or with overt misalignment of the surface detection algorithm on at least 10% of consecutive A-scans or 15% of cumulative A-scans, and a new image was taken again. The RNFL, cup-to-disc (C/D) ratio, and disc area were retrieved from the OCT results.

### Statistical Analysis

The OPP was determined according to the following formula:

OPP = [2/3 mean AP] – IOP; where, the mean AP (arterial pressure) is 1/3 [SAP – DAP] + DAP. SAP is the systolic arterial pressure and DAP is the diastolic arterial pressure.

The predictive ICP was calculated according to the equation of Xie et al:^[11,12]^

ICP = (0.44 × BMI) + (0.16 × DBP) – (0.18 × age) – 1.91; where, ICP is intracranial pressure (mmHg), BMI is body mass index (kg/m${}^{2}$), DBP is diastolic blood pressure (mmHg), and age input is in years.

The TLPD was calculated as the arithmetic difference between the IOP and ICP (TLPD = IOP – ICP).^[13]^

The sample was stratified into three quantiles according to the optic disc size, that is, the disc area (mm${}^{2}$) as measured by the OCT. Participants' eyes with the same optic disc area were clustered together in the same quantile. The difference between the three groups was compared using the ANOVA test. Data from both eyes were used after applying the suitable correction for inter-eye dependency. Statistical significance was set at *P* < 0.05. All analyses were performed by MedCalc software, version 9.3.7.0 (MedCalc Software bvba, Belgium).

##  RESULTS

The sample consisted of 73 patients who were either diagnosed with POAG or suspected of having POAG. The demographic features of all participants stratified by the optic disc area tertiles are displayed in Table 1. Most patients were White and female. The three groups did not differ in age, gender, or ethnic distribution. After applying the appropriate correction for inter-eye dependency, 140 eyes were included in the final analysis. The clinical features for each eye according to disc area tertile are depicted in Table 2. The groups did not differ in either structural (RNFL thickness and C/D) or functional (MD and PSD) variables. The OPP was lower in patients with smaller disc sizes and higher in patients with average discs. However, the difference did not reach statistical significance (*P* = 0.136). Nevertheless, patients with larger optic disc area presented a higher TLPD as compared to patients with small or average discs (2.4 ± 4.5, 2.8 ± 3.8, and 3.7 ± 4.7 mmHg in the first, second, third tertile, respectively *P* < 0.001).

**Table 1:** Demographic features of the study population according to optic disc size


**Variable**	**First tertile (** ***n*** ** = 25)**	**Second tertile (** ***n*** ** = 23)**	**Third tertile (** ***n*** ** = 25)**	**** ***P*** **-value**
**Age (yr)**	69.2 ± 8.7	64.8 ± 9.8	67.4 ± 9.3	0.563
**Gender (M:F)**	11:14	9:14	10:16	0.982
**Ethnicity**		0.968
*White*	15	14	17	
*Non-white*	10	9	9	
M, male; F, female				

**Table 2:** Clinical characteristics of 140 eyes stratified by optic disc size.


**Variable**	**1st tertile (n = 46 eyes)**	**2nd tertile (n = 46 eyes)**	**3rd tertile (n = 48 eyes)**	**** ***P*** **-value**
**Disc area (mm${}^{2}$)**	1.8 ± 0.2	2.2 ± 0.1	2.8 ± 0.3	<.001
**RNFL (µm)**	74.5 ± 16.9	75.6 ± 15.8	79.4 ± 17.4	0.330
**Vertical C/D**	0.78	0.84	0.85	0.08
**MD (dB)**	-12.0 ± 8.7	-13.3 ± 9.4	-11.1 ± 7.5	0.961
**PSD (dB)**	6.9 ± 3.6	6.4 ± 3.3	7.1 ± 4.1	0.590
**OPP (mmHg)**	50.9 ± 7.2	55.2 ± 13.6	51.6 ± 10.0	0.136
**TLPD (mmHg)**	2.8 ± 3.8	2.4 ± 4.5	3.7 ± 4.7	<.001
RNFL, average retinal nerve fiber layer thickness; C/D, median cup to disc ratio; MD, mean deviation; PSD, pattern standard deviation; OPP, ocular perfusion pressure; TLPD, translaminar pressure difference				

##  DISCUSSION

In this observational study, glaucoma patients with larger optic discs presented higher TLPD as compared to patients with smaller optic discs. To the best of our knowledge, this was the first study to evaluate the TLPD according to the optic disc size.

Differences in the size of the optic discs are associated with specific anatomical tissues variation of the RNFL and the optic nerve. These disc size-dependent variations may affect the risk and susceptibility to glaucoma.^[10]^ Some of the structural features observed in large optic discs include a proportionally larger number of nerve fibers, a larger neural rim area, a higher cup-to-disk ratio, and a larger and more numerous pores in the lamina cribrosa.^[14,15,16,17,18,19,20]^

The optic nerve head is located in an area between the high-pressure intraocular space and low-pressure subarachnoid space. Hence, the pressure imbalance between these two spaces can cause damage to the retinal ganglion cell axons that pass through the lamina cribrosa pores.^[21,22,23]^ The pressure difference across the lamina cribrosa (IOP minus ICP) is the translaminar pressure gradient.^[24]^ On physiological grounds, the mean IOP is meagerly higher than the mean ICP, which results in a small posteriorly directed translaminar pressure gradient difference of approximately 4 mmHg.^[25]^ An IOP within statistically normal limits in conjunction with a low ICP produce the same pressure gradient across the lámina cribosa (LC) as a high IOP in conjunction with a normal ICP.^[26]^ Changes in the TLPD may cause pathological dysfunction and optic nerve damage attributable to alterations in axonal transportation, LC deformation, changes in blood flow or even all of them in combination.^[2,3][21,22,23][27][28]^ A higher IOP, lower ICP, and larger TLPD correlates with enlargement in the C/D ratio and reduction in RNFL thickness.^[2,29]^ In our study, patients with a larger optic disc area presented with a higher TLPD. For these patients the ganglion cell axons could be more exposed to this pressure gradient. Thus, patients with larger optic discs may be more vulnerable to IOP insults, without simultaneous influence of OPP which did not differ among the three groups. As such, patients with larger discs would be more likely to have glaucoma than patients with smaller optic discs. Interestingly, in cases of progressive optic neuropathies, the optic nerve fiber counts and the anatomic reserve capability are higher in eyes with large optic heads than those with smaller optic discs.^[14]^ Moreover, discs >4.4 mm${}^{2}$ have an augmented number of cilioretinal arteries, which relates to the size of the optic disc area.^[30]^ These characteristics may thwart against the TLPD insult and can work as a compensatory effect.

Recently, Baneke et al have defined the strain in the LC as the function of TLPD times the square of its diameter divided by the square of its thickness [LC stress = (IOP – ICP). LC radius${}^{2}$/LC thickness${}^{2}$].^[31]^ In this model, the TLPD and disc size (radius) were considered as two independent variables of LC stress. If that is the case, larger discs would be vulnerable not only by its anatomic enlarged area but also to a larger TLPD.

A possible association between glaucoma and decreased OPP was demonstrated in several previous studies.^[32,33,34,35,36,37]^ In contrast, the Beijing Eye Study did not find a clear association between the OPP and the prevalence of glaucoma.^[38]^ In this study, the OPP did not vary according to the optic disc size. Thus, the vascular insult should be the same for all discs, regardless of the disc size, and the higher TLPD could be an isolated aggravating factor and independent of the OPP.

This study has one important limitation. The measurement of ICP was not performed by the traditional method using lumbar puncture which is an invasive examination, with the risks of spinal cord injury. For ethical reasons, it was not performed for the study purposes without specific medical indications. The estimated ICP was calculated using a mathematical formula based on the BMI and BP values developed in a population study of Chinese individuals.^[11]^ It is not certain how different the calculated ICP is from the actual one measured by lumbar puncture. ICP is not only influenced by circadian rhythm, but also by changes in posture, position, and pressure fluctuations in other compartments as in respiratory effort and blood pressure pulsations.^[39]^ Moreover, the production and resorption of cerebrospinal fluid rate are not linear, particularly at different ICP levels.^[39]^ Hence, a linear prediction model for ICP based on Xie's formula may be inaccurate, especially for pathologic conditions. In POAG cases, hemodynamic disturbances are known comorbidities and ICP regulation has been suggested to be abnormal. Moreover, the Cushing reflex or vasopressor response may be affected in these patients and the ICP estimation on BP variation may be too simplistic. Using such a surrogate measure could be misleading. This is a fundamental drawback to the study methodology which limits the generalizability of the finding. However, this same formula has been used in other large population studies.^[12,40]^ Furthermore, the equation was validated in a cohort of 39 Brazilian patients and showed that the estimated ICP was very close to the measured ICP (95% limits of agreement of –5 to +8 between LP measured and equation-estimated ICP).^[41]^ Moreover, the measurement of ICP by lumbar puncture may be different from the retrobulbar ICP. In general, it is assumed that the lumbar ICP represents the CSF pressure in the optic nerve. However, given the extended length between the lumbar spine and the subarachnoid space of the optic nerve, it is debatable whether this statement is true, particularly in patients with optic nerve sheath diseases and compartmentalization.^[42]^

In summary, this study revealed that the TLPD varies according to the optic disc size and that larger discs tend to have a higher TLPD. Although additional studies are still needed to elucidate the possible role of ICP and OPP in the pathogenesis of glaucoma optic neuropathy, we believe that this study contributes to the acumen on how the optic disc size may be important in the pathogenesis of this disease.

##  Financial Support and Sponsorship

Nil.

##  Conflicts of Interest

The authors declare that they have no conflict of interest.
